# Systems Biology of Meridians, Acupoints, and Chinese Herbs in Disease

**DOI:** 10.1155/2012/372670

**Published:** 2012-10-18

**Authors:** Li-Ling Lin, Ya-Hui Wang, Chi-Yu Lai, Chan-Lao Chau, Guan-Chin Su, Chun-Yi Yang, Shu-Ying Lou, Szu-Kai Chen, Kuan-Hao Hsu, Yen-Ling Lai, Wei-Ming Wu, Jian-Long Huang, Chih-Hsin Liao, Hsueh-Fen Juan

**Affiliations:** ^1^Department of Life Science, National Taiwan University, No. 1, Section 4, Roosevelt Road, Taipei 106, Taiwan; ^2^Graduate Institute of Epidemiology and Preventive Medicine, National Taiwan University, Taipei 100, Taiwan; ^3^Department of Bioenvironmental Systems Engineering, National Taiwan University, Taipei 106, Taiwan; ^4^Institute of Biochemical Sciences, National Taiwan University, Taipei 106, Taiwan; ^5^Institute of Molecular and Cellular Biology, National Taiwan University, No. 1, Section 4, Roosevelt Road, Taipei 106, Taiwan; ^6^Department of Biochemical Science and Technology, National Taiwan University, Taipei 106, Taiwan; ^7^Graduate Institute of Biomedical Electronic and Bioinformatics, National Taiwan University, No. 1, Section 4, Roosevelt Road, Taipei 106, Taiwan

## Abstract

Meridians, acupoints, and Chinese herbs are important components of traditional Chinese medicine (TCM). They have been used for disease treatment and prevention and as alternative and complementary therapies. Systems biology integrates omics data, such as transcriptional, proteomic, and metabolomics data, in order to obtain a more global and complete picture of biological activity. To further understand the existence and functions of the three components above, we reviewed relevant research in the systems biology literature and found many recent studies that indicate the value of acupuncture and Chinese herbs. Acupuncture is useful in pain moderation and relieves various symptoms arising from acute spinal cord injury and acute ischemic stroke. Moreover, Chinese herbal extracts have been linked to wound repair, the alleviation of postmenopausal osteoporosis severity, and anti-tumor effects, among others. Different acupoints, variations in treatment duration, and herbal extracts can be used to alleviate various symptoms and conditions and to regulate biological pathways by altering gene and protein expression. Our paper demonstrates how systems biology has helped to establish a platform for investigating the efficacy of TCM in treating different diseases and improving treatment strategies.

## 1. Introduction 

According to traditional Chinese medicine (TCM), acupoints are linked in a network of meridians running along the surface of the body. The meridian system is a special channel network that consists of skin with a high concentration of nerves, various nociceptive receptors, and deeper connective tissues inside the body [1]. Moreover, “*q*_*i*_” (vital energy) in TCM is transferred by meridians, and its flow around the body can reflect the health status of individuals [2]. Acupoints are special locations in the body where the “*q*_*i*_” of viscera and meridians infuses and effuses. This phenomenon is thought to be similar to how signals are passed through neural networks. Acupoints are also considered reflection points (i.e., points on the body whose reflexes provide diagnostic information) for certain diseases and are the targets for clinical acupuncture [3].

**Figure 1:**
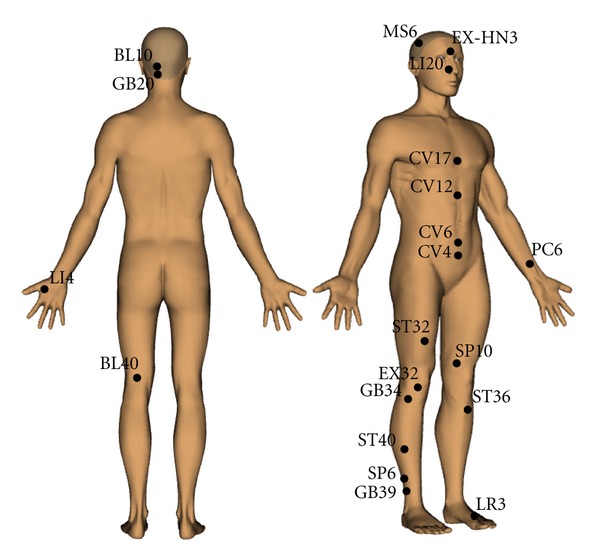
The approximate locations of acupoints on the human body reviewed in this paper. Images are created by Acu3D Ver.1.0.2011.0218.

Acupuncture is an alternative medicine methodology that originated in ancient China. It uses thin metal needles to pierce through skin into acupoints to regulate the flow of *q*_*i*_ around the whole body [4]. Needling at appropriate points can induce effects in locations remote from the insertion site [5]. Many recent brain imaging studies have shown that acupuncture has a specific correlation with the human nervous system. Using functional magnetic resonance imaging (fMRI) to scan the brains of subjects undergoing needling, researchers found that different acupoints corresponded to different cerebral areas and conditioned reactions [3, 6]. Therefore, it has the potential to provide therapy for many diseases. Recent studies have found that electroacupuncture (EA) improved the pathology of motor disorders in a Parkinsonian rat model by restoring homeostasis in the basal ganglia circuit [7, 8]. In other reports, the effects of EA treatment on neuropathic pain [9], acute spinal cord injury [10], acute ischemic stroke [11], and reducing inflammation [12, 13] were also rigorously studied. We review the relevant literature below and have depicted the human acupoints described in this paper in Figure 1.

Another important aspect of TCM is Chinese herbal medicine (CHM), which has been used for thousands of years as a major preventive and therapeutic strategy against disease [14]. Currently, more than 3,200 species of medicinal plants are used in CHM treatments. A fundamental feature of TCM is TCM compound formulas, which are composed of many kinds of herbs and sometimes minerals or animal components, similar to a cocktail therapy [15]. Each TCM compound formula is usually designed to combat specific symptoms and combined with other herbs or prescriptions to tailor to individual needs. Herbal extracts have been investigated for use in treating various diseases and have been used as a complementary or alternative form of medical therapy for cancer patients [16–18]. In other studies that focus on chronic kidney disease [19], neurodegenerative disease [20], and diabetes mellitus [21], Chinese herbs have been reported to alleviate symptoms and mediate signal transduction. 

Systems biology, which combines computational and experimental approaches to analyze complex biological systems, focuses on understanding functional activities from a systems-wide perspective [22]. With the advent of high-throughput global gene expression, proteomics, and metabolomic technologies, systems biology has become a viable approach for improving our knowledge of health and disease [23, 24]. In this paper, we review recent research articles on acupuncture and Chinese herbal therapy performed in conjunction with omics technologies to analyze and investigate the regulatory mechanisms of the treatments and their therapeutic applications.

## 2. Systems Biology and Omics Data

Systems biology is the computational integration of huge datasets to explore biomolecular functional networks [22, 25]. The field offers many approaches and models for searching for biological pathways and predicting their effects and implications [26, 27]. More recently, academic research has focused on developing basic informatics tools that can integrate large quantities of global gene expression, proteomic, and metabolomic data to mimic regulatory networks and cell function [26, 28]. The principle of systems biology is to understand and compare physiology and disease, first from the level of molecular pathways and regulatory networks, then moving up through the cell, tissue, and organ, and finally whole organism levels [29, 30]. It has the potential to provide new concepts to reveal unknown functions at all levels of the organism being studied. 

Omics data helps to explore the different levels in systems biology from a holistic perspective. The suffix “-omics” is added to the object of study or the level of biological process to form new terms to describe that information—for example, genomics from gene data, proteomics from protein data, metabolomics from metabolic data [31]. With rapid progress in sequencing and computational methods, omics techniques have become powerful tools for researching biological mechanisms and diseases and for interdisciplinary applications, for example, biologics, mathematics, and informatics [32–34]. 

## 3. Genomic Studies of Acupuncture in Diseases

Many individual transcriptional profiles of animals or patients have been mined to search for target molecules of acupuncture treatments. Candidate genes or pathways associated with the protective effect of acupuncture treatments have been revealed through genomic analysis for several diseases and symptoms. We list the acupoints and related symptoms in Table 1.

### 3.1. Analgesia

Acupuncture was reported to reduce pain during surgery in the 1950s [35] and can treat different types of pain in many cases [36, 37]. Particularly, previous reports have indicated a major role for acupoint ST36, whose therapeutic properties include analgesia [38]. Gao et al. compared the hypothalamus transcriptional profiles of rats that responded or did not respond to EA analgesia treatment at ST36 [39]. They found that genes for glutamatergic receptors, ghrelin precursor peptides, the melanocortin 4 receptor, and neuroligin 1 may all be new targets for pain management. Moreover, Chae et al. found 375 genes showing significant variation in response to the analgesic effect of acupuncture (on LI4) [40]. Among these genes, cold shock domain protein A and kruppel-like factor 5 were identified as potential targets for investigating the mechanisms behind acupuncture-induced analgesia. 

### 3.2. Antiaging

In an anti-aging study, Ding et al. analyzed the hippocampus gene expression profiles in senescence-accelerated mice given acupuncture treatment [41]. Following applied acupuncture at the CV17, CV12, CV6, ST36, and SP10 acupoints, the researchers observed eight genes affected by age. They found that acupuncture could completely or partially alter the gene expression of heat-shock proteins (Hsp84 and Hsp86) and Y-box binding protein 1—genes related to oxidative damage—in senescence-accelerated mice, indicating that acupuncture may show promise in retarding aging in mammals.

### 3.3. Hypercholesterolemia

Li and Zhang stimulated C57BL/6j mice, which are used in diabetes and obesity research, at the ST40 acupoint and found that cholesterol 7*α* hydroxylase was upregulated following EA, while sodium taurocholate cotransporting polypeptide was downregulated [42]. Each of these variations in gene expression might alter the balance of cholesterol metabolism and reduce cholesterol by regulating bile salt biosynthesis and flux, respectively [43, 44].

### 3.4. Knee Osteoarthritis

Tan et al. compared the transcriptional profiles of four patients stimulated at the CV4, CV6, ST36, EX32, and GB34 acupoints [45]. They collected samples from the peripheral blood of patients after acupuncture treatments. Among differentially expressed genes involved in the pathways, oxidative phosphorylation (ATP synthesis) was found in all patients. Analysis of profiles with respect the Kyoto Encyclopedia of Genes and Genomes (KEGG) pathway database showed that the majority of differentially expressed genes were associated with seven types of metabolic pathways, particularly the primary, cellular, and energy metabolism pathways. These results showed that acupuncture treatment may regulate gene expression to balance energy metabolism of knee osteoarthritis patients. 

### 3.5. Neuronal Diseases

In a spinal cord injury (SCI) study, EA treatment on rats at four acupoints—ST36, GB39, ST32, and SP6—was seen to restore sensory function by microarray analysis [46]. In this study, they found that extending the EA treatment time for SCI rats could result in more changes in gene expression. After EA stimulation, the gene expression of calcitonin gene-related polypeptide (CGRP) and neuropeptide Y (NPY) were up-regulated and functionally annotated with the recovery of sensory functions. Acupuncture treatment at ST36 has also been reported to reduce neuropathic pain. Using microarray analysis, opioid receptor sigma was one of differentially expressed genes and involved in opioid signaling which has been implicated in neuropathic pain and the analgesic effects of EA at ST36 in neuropathic pain model rats [47].

Parkinson's disease is a neurodegenerative disease caused by the death of dopaminergic neurons [48]. Several studies have performed acupuncture treatments on different acupoints in 1-methyl-4-phenyl-1, 2, 3, 6-tetrahydropyridine-(MPTP-) induced Parkinson's models. These models were used to evaluate the effects of acupuncture treatment at the GB34 and LR3 acupoints by analyzing the transcriptional profiles from cervical spinal cord or brain bilateral striatal tissues [49, 50]. Among the genes downregulated by acupuncture treatment, proplatelet basic protein (Ppbp) was functionally annotated with cytokine-cytokine receptor interaction pathways, and cytotoxic T lymphocyte-associated protein 2 alpha (Ctla2a) was associated with a pathway relevant to Parkinson's disease according to KEGG analysis [49]. Similar results were observed from substantia nigra tissue of MPTP mice following treatment at the GB34 acupoint [51]. In previous studies, myelin basic protein, a major constituent of the axonal myelin sheath, has been reported to be up-regulated in MPTP mice and Parkinson's patients stimulated at the GB34 acupoint [52]. In the bilateral striatal tissue of mice brain following acupuncture at the GB34 and LR3 acupoints, the gene expression levels of gap junction alpha 4 protein (Gja4) and tubulin alpha 8 (Tuba8) were down-regulated and annotated with the cell communication and gap junction pathway, respectively. Furthermore, an up-regulated gene, neurotrophin-3 (Ntf3), was annotated with the mitogen-activated protein kinases (MAPK) signaling pathway [50]. 

### 3.6. Immune Modulation and Allergic Rhinitis

Evidence from mouse models has demonstrated that EA or acupuncture stimulation at ST36 can modulate the immune response [53–55]. Two studies using a 2,4-dinitrophenylated keyhole limpet protein (DNP-KLH) immunized mouse model have shown that gene expression patterns can change in the spleen [55] and hypothalamus [54] after EA treatment. Using Sprague Dawley (SD) rats, Kim et al. found that the genes altered following EA at ST36 play crucial roles in natural killer cell activation in spleen tissue [53]. However, the effects of EA treatments remain diverse, partly because of variation in immune responses when triggered in different types of tissues or models (e.g., DNP-KLH immunized mice versus SD rats). 

Acupuncture treatment has also demonstrated some effectiveness against allergic rhinitis in clinical settings, presumably through immune modulation [56, 57]. Allergic rhinitis occurs when an allergen is inhaled and generates an immune response in an individual. Its symptoms can be reduced by acupuncture treatment at the EX-HN3, LI4, LI20, and ST36 acupoints [56]. The effectiveness of the treatment could be accounted for by modulation of pro- and anti-inflammatory genes observed through microarray analyses of blood samples. Immune responses to allergic rhinitis may vary depending on types of allergen. Shiue et al. have used the Phadiatop (Ph) assay, an effective screening tool that detects a diverse range of allergens, to demonstrate that patients with Ph-positive (+) and Ph-negative (−) allergic rhinitis display different gene expression profiles after acupuncture treatment [57]. A hierarchical clustering analysis revealed that three gene groups—those for active immune response, regulatory T cell differential, and apoptosis—were differentially expressed in the Ph (+) and Ph (−) patients after treatment [57]. These results also indicate the importance of personalized medicine for future investigations. 

## 4. Proteomic Studies of Acupuncture in Diseases

Proteomic technologies, such as two-dimensional electrophoresis (2-DE), can screen for proteins differentially expressed between individuals responsive and nonresponsive to acupuncture treatments. These proteins were identified by various mass spectrometry (MS) techniques, and their related pathways were explored to determine the mechanism of the acupuncture treatments. The results clarify the relationships between different acupoints and functions (Table 1). 

### 4.1. Acute Ischemic Stroke

EA efficacy has been studied by comparing levels of serum proteins in response to EA or drug treatments in acute ischemic stroke patients [11]. Patients were treated with EA at eight acupoints (MS6, BL10, GB20, LI4, PC6, B40, SP6, and ST36) once daily for ten consecutive days. The protein profiles were analyzed using 2-DE; SerpinG1 was up-regulated, while gelsolin, complement component 1 (C1), C3, C4B, and beta-2-glycoprotein I were all down-regulated. Other studies have indicated that platelet C4 expression is associated with acute ischemic stroke by comparing serum proteins from healthy individuals and ischemic stroke patients [58].

### 4.2. Neuronal Diseases

Using matrix-assisted laser desorption/ionization time-of-flight mass spectroscopy (MALDI TOF-MS) and subsequent protein database mining, Li et al. identified fifteen candidate proteins whose expression levels varied with acupuncture treatment for SCI [59]. Among them, annexin A5 (ANXA5) and collapsin response modifier protein 2 (CRMP2) were determined to be beneficial for neuronal survival and axonal regeneration. ANXA5 is a member of the annexin superfamily of calcium- and phospholipid-binding proteins, which are related to apoptosis and inflammation [60]. CRMP2 is a member of the collapsin response mediator protein family and is expressed exclusively in the nervous system, especially during development [61]. Additionally, among up-regulated proteins, heat shock protein beta-1 (HSPB1) has a reported role in cell stress/neuroprotection as well as in axonal regeneration [62]. These results reveal the potential for proteomics research to supplement and guide the treatment of SCI with acupuncture in future research.

Moreover, Sung et al. applied EA at ST36 in a SD rat model, and subsequent 2-DE was used to identify signaling pathways involved in neuropathic pain [9]. Thirty-six differentially expressed proteins were identified in the neuropathic pain model, and the normal expression levels of their corresponding genes could be restored following EA treatment. Furthermore, Jeon et al. performed EA at GB34 in an MPTP mouse model of Parkinson's disease [63]. They observed restoration of behavioral impairment and rescued tyrosine hydroxylase-positive dopaminergic neurodegeneration after the treatment. In previous studies, the expression of myelin basic protein can also be restored to normal levels after EA treatment [63]. Kim et al. performed similar studies at GB34 and GB39 [64]. They identified thirteen differentially expressed proteins using MS, and four of these proteins—cytosolic malate dehydrogenase, munc18-1, hydroxyacylglutathione hydrolase, and cytochrome c oxidase subunit Vb—were restored to normal expression levels following EA treatment. These proteins are involved in cell metabolism, and may reduce MPTP-induced dopaminergic neuronal destruction by decreasing oxidative stress. Taken together, these results suggest that acupuncture treatment is likely to have a neuroprotective effect on neuronal diseases through cell metabolic and nervous tissue developmental pathways, among others. 

## 5. Genomic Studies of Chinese Herbs in Diseases 

Many studies have investigated genes impacted by Chinese herb extracts from transcriptional profiles after treatment and analyzed their functions using pathway databases. These results were used to evaluate whether Chinese herb extracts could be used as a complementary drug to treat specific symptoms. Here, we review Chinese herbal treatment-related studies that incorporated genomic analysis and interpret the efficacy of Chinese herb extracts for various symptoms and conditions (Table 2).

### 5.1. Immunomodulatory Function

It is generally agreed that the fungus *Ganoderma lucidum* contains an abundance of polysaccharides with immunostimulatory properties [65], and these have been investigated using human CD14 (+) (cluster of differentiation 14) derived dendritic cells [66, 67]. Dendritic cells are antigen-presenting cells that play a critical role in the regulation of the adaptive immune response. A comparison of transcriptional profiles from the polysaccharide of *G. lucidum*-treated dendritic cells and untreated dendritic cells showed a decrease in the expression of some phagocytosis-related genes, for example, CD36, CD206, and CD209. The expression of proinflammatory chemokines was increased, that is, chemokine (C-C motif) ligands (CCL) CCL20, CCL5, and CCL19; interleukins (IL) IL-27, IL-23A, IL-12A, and IL-12B; costimulatory molecules CD40, CD54, CD80, and CD86 [67]. Additionally, altered expression levels of CD209, CCL20, CCL5, IL-27, CD54, CD80, and CD86 in cells after treatment with F3 (a polysaccharide fraction extracted from lingzhi) have been observed by Lai et al., with CD209 expression down-regulated proportional to treatment time [66]. Another study investigated the treatment of human CD14+ monocytes with polysaccharide fractions extracted from North American ginseng [68]. The MAPK (extracellular regulated protein kinases-1/2), phosphoinositide-3-kinase, p38, and nuclear factor-kappaB (NF-*κ*B) cascades are key signaling pathways that may trigger immunomodulatory functions, as determined by Ingenuity Pathway Analysis [68]. With regard to other extracts, Cheng et al. reported that the NF-*κ*B pathway was up-regulated in THP-1 by treatment with ethanol extracts of *G. sinense*, but not by *G. lucidum *[69]. Moreover, protosappanin A treatment (an ethanol extract of *Caesalpinia sappan*) has been reported to induce an immunosuppressive effect via the NF-*κ*B pathway in a heart transplantation rat model [70]. 

### 5.2. Wound Repair

Herbal formulas containing extracts of *Astragali radix*, *Rehmanniae radix*, and *Angelica sinensis* show potential therapeutic benefits for wound repair [72]. For example, the formula NF3, which consists of a 2 : 1 ratio of *A. radix* and *R. radix*, has been found to affect cell proliferation, angiogenesis, extracellular matrix formation, and inflammation in the Hs27 skin fibroblast cell line through microarray analysis [72]. Another study in human skin substitutes revealed that SBD.4 extracts of *A. sinensis* possesses skin- and wound-healing activity [73]. After SBD.4 stimulation, the gene expression of collagen XVI and XVII, laminin *γ*-2 and 5, claudin 1 and 4, hyaluronan synthase 3, superoxide dismutase 2, and heparin-binding EGF was up-regulated and ADAM 9 was down-regulated in EpiDermFT skin substitute tissues. The elevation of collagen XVI and XVII can enhance collagen fibril organization and the reduction of ADAM9 may stimulate collagen deposition [89, 90]. These results suggest that these skin- and wound-healing related genes function in cell-substrate junction assembly.

### 5.3. Postmenopausal Osteoporosis

Traditional Chinese herbalists have been treating patients with chronic kidney disease for thousands of years [19]. The effect of an herbal mixture consisting of Herba Epimedii, Fructus Ligustri Lucidi, and Fructus Psoraleae was investigated in aged ovariectomy and calcium deficiency-induced osteoporotic rats [74]. A comparison of the transcriptional profiles between nontreated and herbal formula-treated ovariectomized rats found that some genes specifically activated by the herbal mixture, such as prostaglandin EP3 receptor and osteoprotegerin, were involved in bone remodeling and bone protection. Moreover, they identified the involvement of estrogen-related proteins and suggested that the herbal formula may act like estrogens.

### 5.4. Diabetes Mellitus

Type 2 diabetes mellitus (T2DM) is characterized by insufficient insulin secretion and insulin resistance, such that the liver lacks the ability to regulate glycolysis and gluconeogenesis [91]. Han et al. compared gene expression profiles of C57BL/KsJ-*db/db* mice (insulin-resistant model of insulin-dependent diabetes and obesity) following treatment with compound K (CK), a final metabolite of *Panax ginseng C.A. Meyer* [21]. They found that some differentially expressed genes in liver tissue were associated with glycolysis/gluconeogenesis and pentose phosphate pathways, for example, upregulation of aldolase 2, B isoform and phosphogluconate dehydrogenase for glycolysis and pentose phosphate pathways, and downregulation of fructose bisphosphatase 1 for gluconeogenesis pathways after CK treatment. In adipose tissue, on the other hand, they found differentially expressed genes there was linked to adipocytokine signaling and fatty acid synthesis/metabolism pathways, for example, upregulation of peroxisome proliferator-activated receptor gamma for adipocytokine signaling and fatty acid synthase for fatty acid synthesis pathways after CK treatment. 

Plasma adiponectin, a hormone responsible for increasing neoglucogenesis, is secreted from adipocytes and might play a key role in linking obesity, insulin resistance, and the T2DM syndrome. A higher adiponectin expression has been observed in conjunction with lowered obesity levels in human studies [92]. Furthermore, Fu et al. have found that adiponectin increases total glucose transporter 4 expression, which can aid in the response to insulin at the plasma membrane [93]. These results suggest that CK might be a potential target for antidiabetic drugs.

### 5.5. Antitumor Activity

Chinese herbs have been known to possess anti-tumor activity, and some studies have investigated this functionality and its mechanisms in cancer treatments. F3-treated human leukemia THP-1 cells have been found to undergo apoptosis through death receptor pathways as determined through microarray analysis [71]. Furthermore, F3 induces macrophage-like differentiation by caspase cleavage and p53 activation in THP-1 cells [94]. Another study using a microarray approach determined that 25% of genes regulated by two lingzhi (*G. lucidum* and *G. sinense*) was determined to be similar [69]. *G. sinense* was observed to regulate inflammation and immune response pathways, while *G. lucidum* appeared to increase the expression levels of NF-*κ*B pathway genes. Therefore, lingzhi appears to have efficacy as an anti-tumor agent against THP-1 cells.

Other Chinese herbs have also been associated with anti-tumor effects [95–101]. Two studies have shown that American ginseng (*Panax quinquefolius *L.) extracts can inhibit tumor growth in HCT-116 [75] and MCF-7 cells [76]. A-kinase (PRKA) and anchor protein 8-like (AKAPA8L) gene expression were up-regulated, and phosphatidylinositol transfer protein alpha (PITPNA) gene expression was down-regulated after ginsenoside Rg3 treatment in HCT-116 cells [75]. The inhibition of the MAPK pathway and the up-regulation of Raf-1 kinase inhibitor protein (RKIP) expression were determined in the ginseng extract of hot water-extracted American ginseng-treated MCF-7 cells [76]. These genes have anticancer potential and are considered to be involved in anti-tumor mechanism of America ginseng. A comparison of transcriptional profiles between mouse macrophage RAW 264.7 cells before and after artemisinin treatments [83] found that the differentially expressed genes were most associated with the nitric oxide, cAMP, and Wnt/beta-catenin pathways. They suggested that the tumor regulation function of artemisinin might arise from its effect on nitric oxide biosynthesis. Nitric oxide has been proved that it can suppress tumorigenesis [102].

In another study, Hara et al. examined eight benzodixoloquinolizine alkaloids extracted from *Coptidis rhizome* and assessed the strength of their antiproliferative activity in eight human pancreatic cancer cell lines [77]. These results indicated that berberine is the major compound behind boosting the anti-proliferative response. However, the anti-tumor effect of berberine isolated from *C. rhizome* was poorer than that of whole *C. rhizome*, suggesting other components of the fungus are to some degree responsible as well [78]. MCF-7 cells treated with *Coptidis* extracts also displayed increased activation of anti-tumor pathways. Two critical anti-tumor cytokines were identified—interferon-*β* and tumor necrosis factor-*α*—and *Coptidis* extracts were also found to induce cell growth arrest and apoptosis [103]. These studies suggest that *C. rhizome *or *Coptidis* extracts are able to inhibit cell proliferation by reducing tumor cell growth and promoting apoptosis. 

### 5.6. Angiogenesis

Some Chinese herbs have been reported to promote angiogenesis. Ginseng refers to both *Panax ginseng *C.A. Meyer and *Panax quinquefolius *L. (Araliaceae), which contain similar components. Rg1, a *P. ginseng *extract, can promote angiogenesis by modulating cytoskeletal-related genes and enhancing endothelial nitric oxide synthase activities in human umbilical vein endothelial cells (HUVEC) [82]. Chan et al. also illustrated that Rg1-induced down-regulation of miR-214 led to an increase in the expression of eNOS in HUVEC through miRNA microarray analysis [104]. Taken together, the findings suggest that Rg1, the major component ginosenoside from *P. ginseng*, can promote angiogenesis in HUVEC.

### 5.7. Cardiovascular Disease

*Salvia miltiorrhiza* is widely used for human cardiovascular disorders in Asia, but the cellular mechanism by which it attenuates the growth of aortic smooth muscle under oxidative stress remains unclear. Salvianolic acid (SAL) or tanshinone (TAN) purified from *S. miltiorrhiza* was used to treat acute myocardial infarction in Wistar rats [85]. SAL decreases the gene expression of apoptosis-related genes at in a later period after ischemia, for example, BCL2 modifying factor (Bmf). TAN decreases the gene expression of intracellular calcium pathways-related genes at an early stage after ischemic injury, for example, voltage-dependent calcium channel alpha 1 (CACNA1). Intracellular calcium and apoptosis pathways have been reported to be associated with ischemic cardiac injury and repair [105, 106]. These results suggest that SAL and TAN could be used to prevent injury and involved in after injury repair of acute myocardial infarction.

### 5.8. Neuronal Diseases

Su et al. compared gene expression profiles of H_2_O_2_-exposed human neuroblastoma SH-SY5Y cells following treatment with paeonol, which is extracted from *Paeonia suffruticosa *[20, 84]. They identified that the extract up-regulated the mature T-cell gene set and found that paeonol was able to reduce H_2_O_2_-induced NF-*κ*B activity. These data indicate that paeonol might have antioxidative related properties and could be used to treat neurodegenerative diseases, for example, Alzheimer's disease [107].

## 6. Proteomic Studies of Chinese Herbs in Diseases

Compared with studies investigating Chinese herbs using genomic analysis, proteomic analytic studies have been performed far less and on fewer herbs. Tumors and convulsive disorders are the major subjects of analysis by proteomic technologies to date. The Chinese herbs used for these conditions and related references are documented in Table 2.

### 6.1. Antitumor Activity

HepG2 liver cancer cells were treated with oridonin extracted from *Isodon rubescens* and analyzed by 2-DE and MALDI-TOF-MS [79]. Proteomic data showed that expression levels of heat shock 70 kDa protein 1, Sti1h, and hnRNP-E1 were altered after treatment; these proteins are associated with apoptosis pathways. An extract from Franquet (Cucurbitaceae), tubeimoside-1 (TBMS1), has also been used as an anticancer treatment [108]. Xu et al. found 15 proteins differentially expressed between TBMS-treated or -untreated HeLa cells through MALDI-TOF-MS analysis [80]. These proteins were associated with mitochondrial dysfunction and ER stress-induced cell death pathways and participated in TBMS1-induced cytotoxicity [80].

The major component of *Rhizoma paridis*, *Rhizoma paridis* total saponin (RPTS), is responsible for the antitumor effects of this herb. Using MALDI-TOF-MS, Cheng et al. identified 15 proteins altered in HepG2 cells differentially expressed between RPTS-treated or untreated HepG2 cells, with most of them implicated in tumor initiation, promotion, and progression [81]. These results suggest that proteomic approaches could be useful tools to elucidate pharmacological mechanisms responsible for anti-cancer drug activities.

Moreover, Hung et al. found that *S. miltiorrhiza* aqueous extract (SMAE) inhibited the proliferation of rat aortic smooth muscle cell line A10 under homocysteine (Hcy)-induced oxidative stress [87, 88]. Furthermore, the intracellular reactive oxygen species concentration significantly decreased in A10 cells after SMAE treatment. Using MALDI-TOF-MS, the researchers suggested that the SMAE-induced inhibition of growth in Hcy-stimulated A10 cells occurred via the PKC/MAPK-dependent pathway [87, 88].

### 6.2. Convulsive Disorders

*Uncaria rhynchophylla* (UR) and its major component, rhynchophylline, have demonstrated effectiveness in treating convulsive disorders [109]. Lo et al. used SD rats with kainic acid (KA)-induced epileptic seizures and treated them with UR [86]. They then analyzed proteomic profiles from the frontal cortex and hippocampus of rat brain tissues and identified proteins differentially expressed between treated and untreated tissue. Macrophage migration inhibitory factor (MIF) and cyclophilin A were down-regulated and restored to normal levels in epileptic seizure rats after UR treatment. MIF has been considered as a counterregulator of normal neuronal actions and increases its expression level to reduce the chronotropic actions in SD rats [110]. Cyclophilin A is a phylogenetically-conserved protein and regulates immunosuppression [111]. These results showed that MIF and cyclophilin A involved in the mechanism of anticonvulsive effect of UR. 

## 7. Metabolomics Studies of Chinese Herbs in Diseases

Another platform for systems biology research is metabolomics, involving the study of targeted small molecule metabolites (<1500 Da). In 1998, the metabolome was first introduced in the elucidation of yeast gene function [112]. However, the application of this concept can be traced back to the development of traditional medicine; while metabonomics chemically tracks metabolites in urine, feces, and so forth, traditional medicine used color, smell, and taste to facilitate diagnoses. Over genomics and proteomics, metabolomics can provide a more solid link between genotype and phenotype. 

### 7.1. NMR-Based Metabolomics Analysis

NMR-based metabolomics is an attractive method for the study of medicinal herb efficacy on disease symptoms. Using this method, nonselective and comprehensive analysis was performed on ginkgo extracts [113]. Moreover, Zhang et al. found that ginkgo extracts have multidirectional lipid-lowering effects on the rat metabonome [114]. They suggested that ginkgo extracts possess metabolomic functions, including limitation of cholesterol absorption, inactivation of HMGCoA, and favorable regulation of essential polyunsaturated fatty acid profiles [114]. 

### 7.2. Liquid Chromatography (LC)-TOF MS-Based Metabolomics

LC-MS-based metabolomics have been explored in many studies to date. Tan et al. performed LC-TOF MS to investigate the metabolites of aconitum alkaloids in rat urine after oral administration of aconite root extracts [115]. They found 10 metabolites and 24 parent components and suggested that metabolomic approaches may prove useful in exploring the efficacy of CHM. Moreover, Yan et al. investigated the antiaging effect of flavones contained in *Epimedium* extract in rats through LC-MS [116]. They were able to identify differences in multiple changed age-related metabolites in serum, including carnosine, ergothioneine, unsaturated fatty acids, saturated fatty acids, and nucleotides. The expression levels of these age-related proteins were restored to levels found in younger rats after *Epimedium* treatment.

Terpenoids, a group of important secondary metabolites in plants, are found in *Ganoderma* sp., which have high cytotoxic and anti-tumor activity. Ganoderiol F, a tetracyclic triterpene, has been analyzed by LC/MS/MS and administered to rats for metabolomics and pharmacokinetics experiments [117]. Analysis of the metabolites of ganoderiol F by HPLC/MS/MS from orally or i.v.-treated rats showed good viability and low acute toxicity. According to these reports, ganoderiol F may show potential as an anti-cancer drug.

## 8. Summary and Future Perspectives 

**Figure 2:**
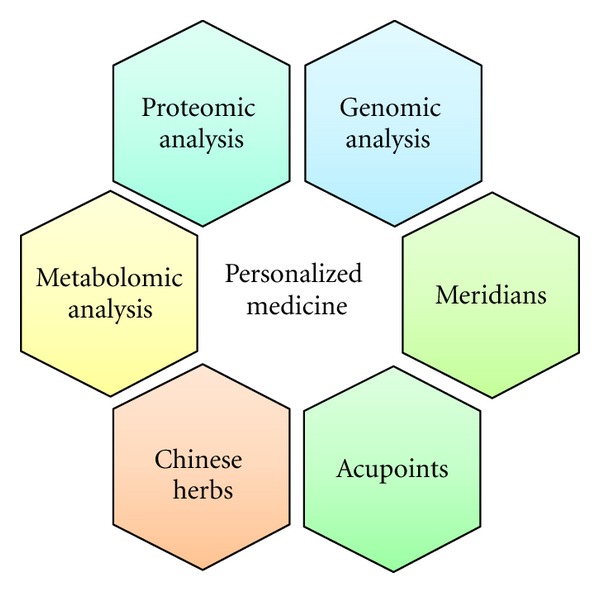
The composition of “personalized medicine in TCM.” Combining genomic, proteomic, and metabolomic information can provide more comprehensive strategies for TCM therapies.

In this paper, we described and discussed omics research to date combined with acupuncture or CHM, performed at the systems biology level. We found that the ST36 acupoint is the most widely used acupoints, and that it has multiple therapeutic functions and targets, including spinal cord injury [46], allergic rhinitis [56], analgesia [38], neuropathic pain [9, 47], antiaging [41], knee osteoarthritis [45], and acute ischemic stroke [11]. Different from other therapies, acupuncture treatments show variation in the transcriptional profiles and perceived effect of their subjects, whether rats [39] or humans [40]. Gao et al. reported that approximately 30% of rats demonstrated no analgesic effects during EA [39]. Similarly, Chae et al. reported that 40% of their participants felt only low analgesic effects during acupuncture, an observation that is more likely caused by genetic variation rather than differences in psychology [40]. Individual variance in treatment response is becoming an important issue when deciding whether it is suitable or not to administer acupuncture therapy. Based on this paper results, we suggest that system biology approaches can be performed to construct exhaustive clinical data of patients with acupuncture or CHM treatments, for example, up-regulated or down-regulated genes of Ph (+) or Ph (−) allergic rhinitis patients with acupuncture treatments, and provide useful information for improving future therapeutic strategies. In term of acupuncture treatment, one acupoint commonly used for different symptoms, for example, LI4 acupoint could be performed for allergic rhinitis, analgesia, and acute ischemic stroke. Compared with acupuncture treatments, Chinese herbal treatments are more often investigated for their effectiveness on specific diseases, and studies focus on the pharmacological mechanisms of different herbal extracts, for example, benzodixoloquinolizine alkaloids has anti-tumor activity [77] or CK has anti-diabetic efficacy [21]. In summary, we suggest appropriate therapies—whether acupuncture, TCM, or a combination—can be personalized to individuals through analyzing their transcriptional or proteomic profiles (Figure 2). “Personalized medicine in TCM” can be developed even further and provide important information for therapeutic strategies in managing various diseases and conditions.

## Figures and Tables

**Table 1:** List of the acupuncture points (acupoints) reviewed in this paper.

Acupoint Chinese name	Acupoint name	Function/target	References
Weizhong	BL40	Acute ischemic stroke	[11]
Tianzhu	BL10	Acute ischemic stroke	[11]
Guanyuan	CV4	Knee osteoarthritis	[45]
Qihai	CV6	Antiaging, knee osteoarthritis	[41, 45]
Zhongwan	CV12	Antiaging	[41]
Danzhong	CV17	Antiaging	[41]
Yintang	EX-HN3	Allergic rhinitis	[56]
Xiyan	EX32	Knee osteoarthritis	[45]
Fengchi	GB20	Acute ischemic stroke	[11]
Yanglingquan	GB34	Knee osteoarthritis, Parkinson's disease	[45, 49–52, 63, 64]
Xuanzhong	GB39	Spinal cord injury, Parkinson's disease,	[46, 64]
Hegu	LI4	Allergic rhinitis, analgesia, and acute ischemic stroke	[11, 40, 56]
Yingxiang	LI20	Allergic rhinitis	[46, 56]
Taichong	LR3	Parkinson's disease	[49, 50]
Motor Area	MS6	Acute ischemic stroke	[11]
Neiguan	PC6	Acute ischemic stroke	[11]
Sanyinjiao	SP6	Spinal cord injury, acute ischemic stroke	[11, 46]
Xuehai	SP10	Anti-aging	[41]
Futu	ST32	Spinal cord injury	[46]
Zusanli	ST36	Neuropathic pain, spinal cord injury, immune modulation, allergic rhinitis, analgesia, anti-aging, knee osteoarthritis, and acute ischemic stroke	[9, 11, 38–41, 45–47, 53–56]
Fenglong	ST40	Hypercholesterolemia	[42]

**Table 2:** List of Chinese herbal studies that have incorporated genomic and proteomic analysis.

Authors	Herb name	Extracts	Common functions
Sliva [65], Lai et al. [66], Lin et al. [67], Cheng et al. [71]	*G. lucidum *(*lingzhi*)	Polysaccharide fraction/F3	Immunomodulatory, antitumor activity
Cheng et al. [69]	*G. sinense *(*lingzhi*)	Ethanol extracts	Immunomodulatory
Wu et al. [70]	*C. sappan *	Protosappanin A	Immunomodulatory
Zhang et al. [72]	A. Radix and R. Radix	NF3 (A. Radix and R. Radix in the ratio of 2 : 1)	Wound repair
Zhao et al. [73]	*A. Sinensis *	SBD.4	Wound repair
Sun et al. [74]	Herba Epimedii, Fructus Ligustri Lucidi, and Fructus Psoraleae		Postmenopausal osteoporosis
Han et al. [21]	C.A. Meyer	CK	Diabetes mellitus
Luo et al. [75], King and Murphy [76]	American *ginseng * (*P. quinquefolius *L.)	Ginsenoside Rg3/ginseng extracts	Antitumor activity
Hara et al. [77], Iizuka et al. [78]	*C. rhizome *	Benzodixoloquinolizine alkaloids/berberine	Anti-tumor activity
Wang et al. [79]	*L. rubescens *	Oridonin	Anti-tumor activity
Xu et al. [80]	Franquet	TBMS1	Anti-tumor activity
Cheng et al. [81]	*R. paridis *	RPTS	Anti-tumor activity
Yue et al. [82]	*P. ginseng *	Rg1	Angiogenesis
Konkimalla et al. [83]	Artemisinin		Nitric oxide biosynthesis
Su et al. [20, 84]	*P. suffruticosa *	Paeonol	Neurodegenerative disease
Wang et al. [85]	*S. miltiorrhiza *	SAL or TAN	Acute myocardial infarction
Lo et al. [86]	*U. rhynchophylla *		Convulsive disorders
Hung et al. [87, 88]	*S. miltiorrhiza *	SMAE	Cardiovascular disorder
